# AgDscam, a Hypervariable Immunoglobulin Domain-Containing Receptor of the Anopheles gambiae Innate Immune System

**DOI:** 10.1371/journal.pbio.0040229

**Published:** 2006-06-20

**Authors:** Yuemei Dong, Harry E Taylor, George Dimopoulos

**Affiliations:** **1**W. Harry Feinstone Department of Molecular Microbiology and Immunology, Bloomberg School of Public Health, Johns Hopkins University, Baltimore, Maryland, United States of America; Stanford UniversityUnited States of America

## Abstract

Activation of the insect innate immune system is dependent on a limited number of pattern recognition receptors (PRRs) capable of interacting with pathogen-associated molecular pattern. Here we report a novel role of an alternatively spliced hypervariable immunoglobulin domain-encoding gene, *Dscam,* in generating a broad range of PRRs implicated in immune defense in the malaria vector Anopheles gambiae. The mosquito Down syndrome cell adhesion molecule gene, *AgDscam,* has a complex genome organization with 101 exons that can produce over 31,000 potential alternative splice forms with different combinations of adhesive domains and interaction specificities. *AgDscam* responds to infection by producing pathogen challenge-specific splice form repertoires. Transient silencing of *AgDscam* compromises the mosquito's resistance to infections with bacteria and the malaria parasite *Plasmodium*. AgDscam is mediating phagocytosis of bacteria with which it can associate and defend against in a splice form–specific manner. AgDscam is a hypervariable PRR of the A. gambiae innate immune system.

## Abstract 2

AgDscam has 101 exons that can produce more than 31,000 potential alternative splice forms. Specific splice forms produced in response to infection by a given pathogen contribute to the mosquito's resistance against pathogens.

## Introduction

The insect's innate immune system is activated upon specific recognition of pathogen-associated molecular patterns by germline-encoded pattern recognition receptors (PRRs). Upon recognition, these receptors can either directly mediate microbial killing through mechanisms such as encapsulation and phagocytosis, or indirectly through the induction of intracellular signaling pathways that control transcription of effector genes [ 1– 8]. Despite the exposure to a very broad range of pathogens, the insect innate immune system lacks the type and specificity of adaptive capacity and memory that are characteristics for the vertebrate immune system. However, several studies have indicated that the innate immune systems of invertebrates may have some type of memory and adaptive ability [ 9– 13]. A certain degree of anti-pathogen defense specificity in insects is achieved through the differential activation of immune response pathways by pathogen class-specific PRRs [ 4, 14]. Disease-transmitting mosquitoes have to cope with a diverse range of potential pathogens due to their complex life cycle, diverse breeding habitats, and hematophagy. It is intriguing that the approximately 150 predicted PRR genes found in the Anopheles gambiae genome can cope with this broad microbial exposure [ 15]. The vertebrate immune surveillance system is capable of discriminating between a similarly broad microbial spectrum through a very large and diverse pattern recognition repertoire, which is provided by V(D)J recombination and somatic hyper-mutation of the antibody immunoglobulin (Ig) domains. Insects do not produce antibodies, but the Drosophila melanogaster and A. gambiae genomes contain some 140–150 Ig domain protein- encoding genes that have mostly been studied in the context of neuronal guidance and also recently for implication in immunity [ 16– 18]. One of the D. melanogaster Ig gene superfamily members, Down syndrome cell adhesion molecule gene *Dscam,* can produce some 38,016 different alternative splice forms through alternative splicing of 95 variable exons [ 19]. Dscam is implicated in neuronal wiring through axon guidance, and alternative splicing enables *Dscam* to produce a broad repertoire of molecules containing variable Ig domain combinations with different specificities in recognition and binding. The significance of this diversity for Dscam function is not clear. In the D. melanogaster olfactory system, the diversity of Dscam has been proposed to provide a mechanism by which dendrites of the same neuron can avoid each other as they elaborate their receptive fields. This mechanism appears to depend on Dscam's capacity to engage in homophilic interactions and is likely to be independent of the actual Dscam sequence itself [ 20]. The vertebrate *Dscam* has been linked to the Down syndrome, but either of the two human Dscam paralogs studied displays a significant degree of alternative splicing. The zebrafish Dscam was recently shown to be essential for cell migration. In the fruit fly, *Dscam* is highly expressed in cell types that play major roles in the fly's innate immune system and have no known functions in the nervous system, suggesting it may have multiple functions [ 17– 26]. A recent study has reported high levels of Dscam in the fruit fly haemolymph and S2 cell-conditioned medium, and RNA interference (RNAi)-mediated depletion of Dscam was shown to impair the haemocyte's capacity of phagocytosing bacteria [ 18]. Here we present evidence of the function of the A. gambiae Dscam, AgDscam, as a versatile PRR, capable of producing pathogen-specific splice form repertoires upon immune challenge.

## Results/Discussion

### Genome Organization and Exon Sequence Divergence Patterns

**Figure 1:**
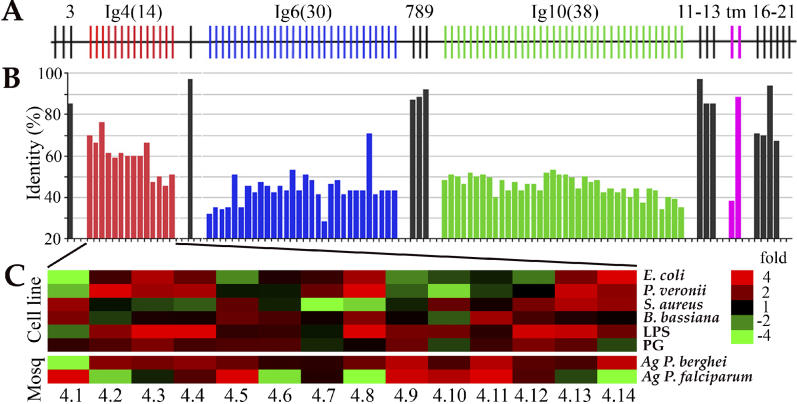
AgDscam Gene Organization and Infection-Responsive Splicing (A) The variable Ig domain exon cassettes 4, 6, and 10 are displayed as Ig4(14) (red), Ig6(30) (blue), and Ig10(38) (green), each containing 14, 30, and 38 exons, respectively. The transmembrane domain exons (tm) 11–13 are displayed in pink, and non-spliced exons are displayed in black. (B) Percentage identity of AgDscam exons to the D. melanogaster Dscam exons at the amino acid level is displayed. Phylogenetic relations between AgDscam Ig exons are presented in Figure S1. (C) Differences in *AgDscam* exon 4 transcript representations between bacterially- *(E. coli, P. veronii, S. aureus),* fungus- *(B. bassiana),* LPS-, and PG-challenged cell line Sua5B and a non-challenged cell line at 12 h after challenge, and *Plasmodium- (P. berghei* and *P. falciparum)* infected midguts (Mosq) and non-infected blood-fed midguts at 24 h after ingestion. The numbers (“4.1”–“4.14”) indicate the individual exons of exon cassette 4. Expression was determined with RTqPCR analyses in three replica assays. The fold difference in exon representation (ratio) between challenged and naive samples from three replicates were determined in normalized cDNAs, and expression ratios are displayed in a color scheme where red indicates a higher representation and green indicates a lower representation of exons in challenged samples compared to naïve samples. Black was indicative of a lack of infection-responsive regulation. Normalization was done with expression analysis of a non-spliced *AgDscam* exon and a ribosomal S7 gene. The significance of variable exon regulation at a 95% confidence level, the RTqPCR efficiencies for each pair of exon primers, expression data values, and standard errors are presented in Table S1.

Similarly to the D. melanogaster ortholog, *AgDscam* comprises three variable Ig domain exon cassettes, 4, 6, and 10, each consisting of 14, 30, and 38 alternatively spliced Ig domain exons, respectively ( Figure 1A). In theory, *AgDscam* can generate 31,920 alternative splice forms ( Figure 1B). The majority of the non-alternatively spliced exons are highly conserved between the A. gambiae and *D. melanogaster Dscam,* ranging from 70%–95% at the amino acid level while the spliced Ig domain exons have only 30%–70% homology ( Figure 1B) [ 27]. This pattern suggests that the constitutive and alternative exons are under different functional constraints. Constitutive exons, such as the intracellular exons 16–21, might be implicated in more conserved functions, such as the regulation of downstream signaling pathways that regulate the actin filament necessary for axon guidance and cell migration [ 26– 29]. In contrast, the selective pressure causing the high degree of Ig domain sequence divergence must relate to profound differences in the lifestyles and environmental exposure of the two insects.

The higher degree of conservation between exons of one species compared to exons between species has been suggested to reflect a functional constraint of maintaining the ability to engage in homophilic interactions. Similarly to the *D. melanogaster Dscam,* exons within each of the *AgDscam* exon cassettes are more similar than between exon cassettes, and proximal exons within each cassette are more conserved than distal exons ( Figures 1B and S1) [ 27]. The differential sequence divergence pattern of *Dscam'*s spliced adhesive Ig domain exons and the non-spliced exons, between A. gambiae and *D. melanogaster,* resembles the sequence divergence patterns of their immune gene repertoires; adhesive PRRs have diverged significantly to cope with the different microbial exposures while components of the intracellular signaling pathways Toll and Imd are highly conserved [ 15].

### Infection-Responsive Alternative Splicing

Regulation of A. gambiae immune responses occurs to a significant degree at the transcriptional level [ 28]. *AgDscam* was not responding to infection by an overall increase of its transcripts, but by profound changes of its variable exon representation through alternative splicing. Challenge of the haemocyte-like immune competent cell line Sua5B, with bacteria-, fungi-, and pathogen-associated surface molecules was followed by rapid and specific changes of exon usage in an acute phase response manner ( Figure 1C and Table S1) [ 4, 30]. Exposure to different elicitors elicited production of different splice form repertoires that are characterized by over- or under- representation of specific Ig domain exon-containing splice forms. Hence, challenge with different pathogens resulted in the production of AgDscam molecules with different adhesive characteristics and interaction specificity. For instance, challenge with Escherichia coli resulted in the predominant production of AgDscam splice forms containing exon 4.8, 4.3, and 4.14, as well as, but at a lower proportion, splice forms with exons 4.4 and 4.13. Challenge with Staphylococcus aureus induced transcription of *AgDscam* splice forms containing exon 4.1, 4.13, and 4.14, and to a lesser extent, of exons 4.10 and 4.12. Cells challenged with the two Gram-negative bacteria, *E. coli* and *Pseudomona veronii,* generated quite similar expression patterns that correlated by a coefficient of 0.80 (Pearson's *r* = 0.80, *n* = 14, *p* = 0.0007). Exposure of cells to the bacterial surface molecules peptidoglycan (PG) (characteristic for Gram-positive bacteria) and lipopolysaccharide (LPS) (characteristic for Gram-negative bacteria) showed only some degree of correlation with the splice form repertoires that were induced by the Gram-positive S. aureus or Gram-negative *E. coli,* respectively. This may be attributed to the influence of other elicitors present on the bacterial surface and possibly also the common PG contaminants of LPS preparations [ 31, 32]. The stronger induction of specific splice forms by these purified elicitors is most likely attributed to their relative higher concentration compared to cells challenged with bacteria. The complexity of pathogen-induced splice form repertoires could be fairly high considering the additional alternatively spliced Ig exon cassettes 6 and 10 that are not assayed in this study [ 33]. Previous studies have shown an elicitor dosage dependence of immune gene regulation in an A. gambiae immune competence cell line; a challenge with a lower dose resulted in a smaller magnitude of regulation [ 34]. Challenge of cells with a 10-fold lower dosage (10 ^6^ colony-forming units (CFU)/10 ^6^ cells) of E. coli resulted in a similar but less intense differential splicing pattern (correlation between two dosage treatments were: Pearson's *r* = 0.55, *n* = 14, *p* = 0.0409) ( Figure S2). The infection-responsive splice patterns are persisting up to at least 18 h after E. coli or S. aureus challenge (later time points were not assays) (correlation of splicing regulation between 12- and 18-h time points were: *E. coli:* Pearson's *r* = 0.61, *n* = 14, *p* = 0.0209; *S. aureus:* Pearson's *r* = 0.83, *n* = 14, *p* = 0.0003) ( Figure S2).

Bacteria challenge also triggered alternative splicing of *AgDscam* in the adult mosquitoes (data shown in Figure S2). However, the diverse expression of different *Dscam* splice forms in different tissues and cell types renders the definition of a specific immune challenge-induced splice form repertoire complicated in a complex sample as the whole insect. Different tissues and cell types may differ in their immune gene regulation upon challenge with the same elicitor, and *AgDscam* may in some cell types respond to challenge in a non-immunity–related context [ 18, 22, 35]. The correlation of expression pattern in cell lines and adult mosquitoes upon E. coli challenge was quite significant (Pearson's *r* = 0.73, *n* = 14, *p* = 0.003), but not for S. aureus challenge (Pearson's *r* = 0.4166, *n* = 14, *p* = 0.1384). Upon *Plasmodium* invasion of the midgut epithelium, the *AgDscam* gene responded by changing its transcript exon repertoires in that tissue ( Figure 1C). *AgDscam* regulation in the *Plasmodium*-infected midgut may reflect both immune response to the parasite and the profound midgut epithelial cell reorganizations taking place during the expulsion of parasite-invaded apoptotic cells [ 36]. Interestingly, invasion by the two different parasite species, Plasmodium berghei and *Plasmodium falciparum,* induced quite different *AgDscam* splice form repertoires. Transcript responses of other immune genes to the two parasite species have been shown to be quite diverse in previous studies [ 37]. Little is known about infection-responsive regulation of splicing in general; D. melanogaster and A. gambiae peptidoglycan recognition protein produced different splice forms upon immune challenge, and analyses of infection-responsive genes in the sea urchin identified several splicing factors [ 15, 38– 41]. Functional dissection of an innate immune response by a genome-wide RNAi screen in *Drosophila* revealed regulation of several splicing factors in response to LPS exposure, but the implicated pathways remain unknown [ 42].

### AgDscam Is a Determinant of Resistance to Bacteria Infection

**Figure 2:**
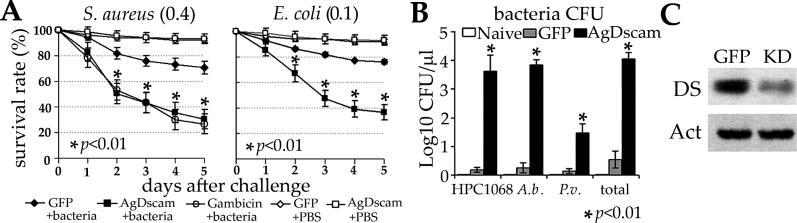
AgDscam Is Implicated in Anti-Bacterial Defense (A) RNAi-mediated depletion of AgDscam with a *dsRNA* fragment complementary to a non-spliced *AgDscam* exon resulted in decreased mosquito survival after challenge with S. aureus and E. coli compared to the challenged control *GFP dsRNA*-treated (GFP) mosquitoes. AgDscam *dsRNA-*treated mosquitoes with a significant decrease in survival compared to *GFP dsRNA-*treated (GFP) mosquitoes, according to a 2-way ANOVA ( *p <* 0.01), are indicated with asterisks. A Kaplan Meier survival analysis of *GFP dsRNA-* and AgDscam *dsRNA-*treated mosquitoes is presented in Figure S3. PBS-injected *GFP dsRNA-* and AgDscam *dsRNA-*treated mosquitoes (“GFP + PBS” and “AgDscam + PBS”) did not exhibit significant mortality. The survival rates of *AgDscam dsRNA-*treated mosquitoes and *Gambicin dsRNA*-treated mosquitoes after injection with Gram-positive bacteria *(S. aureus)* were similar. (B) RNAi-mediated depletion of AgDscam in absence of experimental bacterial challenge resulted in a proliferation of opportunistic bacteria in the mosquito haemolymph at 4 d after *dsRNA* injection, compared to non-treated mosquitoes (naïve) and control *GFP dsRNA-*treated mosquitoes (GFP). The proliferating bacterial species, capable of growing on LB agar, were determined as species closely related to *Bacterium* HPC1068, *Asaia bogorensis (A.b*. *),* and *P. veronii (P.v.).* The total number of bacteria isolated from the haemolymph is indicated with a separate bar (total). (C) Western analysis of AgDscam RNAi-treated 4-d-old female mosquitoes (KD) (mosquito) showed decreased AgDscam protein (DS) by approximately 50% compared to *dsGFP*-treated control (GFP). Total protein was extracted 4 d after *dsRNA* injection and normalized for equal actin (Act) content.

In adult female mosquitoes, *AgDscam* gene silencing through *dsRNA* targeting of a non-spliced exon resulted in about 80% depletion of the *AgDscam* mRNA and 50% depletion of the AgDscam protein in the whole mosquito ( Figure 2C); and impaired the mosquito's capacity to defend against experimental and opportunistic microbial infections ( Figure 2A and 2B) [ 43]. A significant proportion of AgDscam is likely to be expressed in non-immune–competent tissues and cell types that are less accessible to the injected dsRNA. AgDscam's and other immune protein's antimicrobial and anti- *Plasmodium* function are more likely to be carried out by hemocytes that are more susceptible to RNAi gene silencing than other tissues and cell types [ 18, 44]. The mortality of *AgDscam* gene-silenced mosquitoes was assessed daily for 6 d after Gram-negative bacteria *(E. coli)* and Gram-positive bacteria *(S. aureus)* challenge. The survival rates were also subjected to a Kaplan-Meier survival analysis that showed the survival rate of *AgDscam* gene-silenced mosquitoes, after challenge with either E. coli or *S. aureus,* was significantly lower than that of control *GFP dsRNA*-treated mosquitoes challenged with the same pathogens over a 6-d period ( *p* < 0.0001) ( Figure S3). The effect of *AgDscam* gene silencing on survival after S. aureus infection was comparable to that of the antimicrobial peptide, Gambicin gene silencing, which served as a positive control ( Figure 2A) [ 45]. An established positive control for E. coli challenge was not available.

Similarly to vertebrates, the insect innate immune system is continuously defending against opportunistic microbes. Depletion of AgDscam through gene silencing resulted in a profound proliferation of microbes within the mosquito haemolymph, even in absence of experimental challenge. Sequence analyses of the 16s ribosomal genes from the proliferating bacteria suggested close phylogenetic relation to the three Gram-negative bacteria species *Bacterium* HPC1068 (89%), Asaia bogorensis (97%), and P. veronii (99%). These bacteria increased all together by 4 × Log _10_ CFUs per microliter in the *AgDscam*-silenced mosquito haemolymph at 3 d after *dsRNA* injection ( Figure 2B). Therefore, *AgDscam* is an essential factor of mosquito immune defense against bacteria. Most likely, other species of opportunistic bacteria also proliferated in *AgDscam*-silenced mosquitoes but were not detected with the utilized culturing method.

### AgDscam Is a Determinant of Resistance to *Plasmodium* Infection

**Figure 3:**
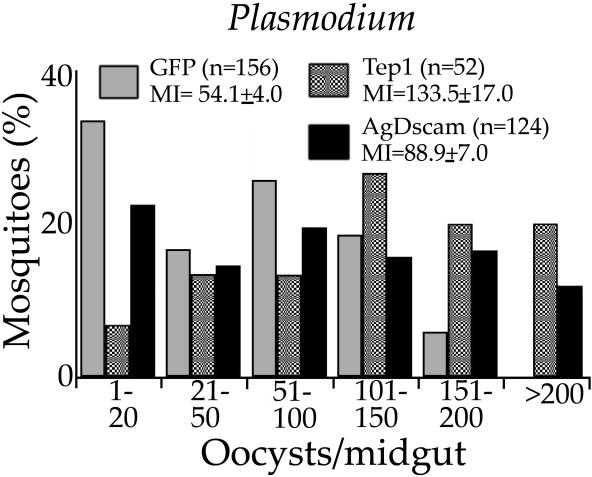
AgDscam Is Implicated in Anti- *Plasmodium* Defense RNAi-mediated depletion of AgDscam from adult female mosquitoes resulted in increased permissiveness to P. berghei infection, as indicated by a 59%–99% increase in oocysts numbers in four independent assays. The figure presents the frequency distributions of oocysts pooled from four independent assays where MI indicates mean intensity of infection (oocysts number) plus/minus standard error, and *n* indicates the total number of mosquitoes in each experiment. Infection levels in *AgDscam*-silenced mosquitoes were comparable to the positive control *Tep1 dsRNA*-treated mosquitoes [ 44].

RNAi-mediated *AgDscam* depletion in mosquitoes fed on mice infected with *GFP-*expressing P. berghei parasites resulted in a statistically significant overall 65% increase of oocysts numbers on the midguts and a larger proportion of mosquitoes with exceptionally high oocysts counts at 13 d after feeding ( Figures 3 and Figure S4) [ 46, 47]. AgDscam is affecting *Plasmodium* development either prior to or during the ookinetes migration through the epithelial cells, or at the stage when ookinetes round up to form oocysts on the basal side of the midgut. The effect of AgDscam gene silencing on *Plasmodium* development was approximately 67% of that recorded for gene silencing of the highly potent anti- *Plasmodium* factor Tep1 in the same experimental mosquito *Plasmodium* system [ 44]. The assayed effect of AgDscam gene silencing on *Plasmodium* development is likely to be significantly reduced by the concomitant up-regulation of other anti- *Plasmodium* immune molecules as a result of the parallel proliferation of the microbial flora in the haemolymph ( Figure 2B) [ 48]. The mosquito's immune responses to bacteria and *Plasmodium* infection have been shown to be significantly overlapping [ 15] (Dimopoulos lab, unpublished data).

### AgDscam Is Implicated in Phagocytosis of Bacteria

**Figure 4:**
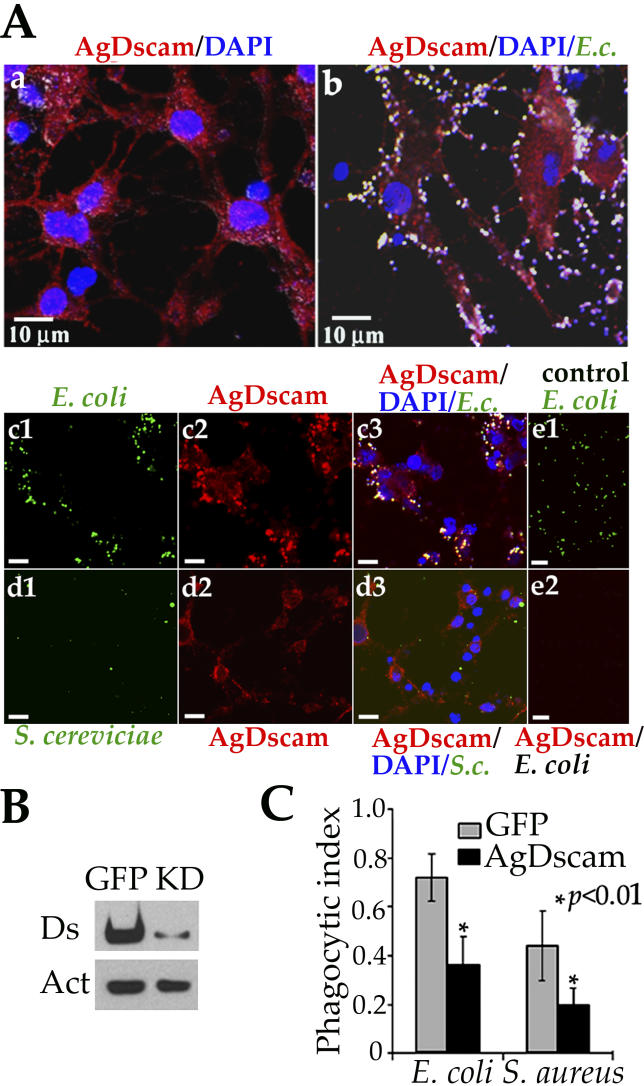
AgDscam Is Implicated in Phagocytosis of Bacteria (A) AgDscam distribution in DAPI-stained Sua5B cells and co-localization with FITC-labeled bacteria [ 30]. In this figure, (a) AgDscam (red) was evenly distributed on the cell surface of non-challenged cells. (b, c1–c3) FITC-labeled E. coli (green, c1) that were co-incubated with the cell line co-localized with AgDscam (red, c2; white, b). Co-localization is indicated in white (b and c3) where cell nuclei were stained with DAPI (blue). (d1–d3) Incubation of FITC-labeled S. cerevisiae (d1) with the same cell line (d2) did not result in phagocytosis and co-localization with AgDscam (d3). S. cerevisiae did not adhere to the cells. Controls: FITC-labeled E. coli (e1) did not interact with the AgDScam antibody or the secondary antibody only (e2). Scale bars: 10 μm. (B) Western analysis of AgDscam (Ds) RNAi-treated Sua5B cell culture (KD) showed decreased AgDscam protein by approximately 82% compared to *dsGFP*-treated control cells (GFP). Total protein was extracted after 6 d of *dsRNA* treatments and samples were normalized for equal actin (Act) content. (C) Phagocytic assay of AgDscam-depleted (AgDscam) and control *GFP dsRNA*-treated Sua5B cells. AgDscam depletion resulted in approximately 50% reduction of phagocytic index for both E. coli and *S. aureus,* which were significantly different from *GFP* controls. Asterisks indicate significant decrease at *p* < 0.01 ( *E. coli: t-*test, *p* = 0.006, df = 36; *S. aureus: t-*test, *p* = 0.008, df = 40). The phagocytic index was calculated as the ratio of the number of immune competent cells containing fluorescent bacteria against the total number of cells in each field. For each assay at least 20 fields were included and data here represented the mean value from three independent assays with standard error bars included.

Both *AgDscam* and the *D. melanogaster Dscam* is expressed in cell lines and haemocytes capable of engulfing and digesting bacteria through the mechanism of phagocytosis, which is dependent on specific recognition of the pathogen by PRRs [ 18, 23, 49] (Dimopoulos and Strand, unpublished data). Interestingly, phagocytosis, which involves signaling cascades that control actin cytoskeletal rearrangements necessary for the cellular extensions used for pathogen engulfment, relies on components that also drive axon guidance and cell migration [ 29, 50]. The D. melanogaster Dscam has been shown to be implicated in phagocytosis of bacteria by larval haemocytes; the phagocytic index decreased by about 45% in Dscam-depleted haemocytes [ 18]. Confocal microscopy showed that AgDscam had an even distribution on the non-challenged A. gambiae immune competent Sua5B cells and became highly concentrated at the site of interaction with E. coli bacteria of challenged cells ( Figure 4A; a,b,c1–c3). Similar results were obtained from immunostaining with S. aureus cells (unpublished data). The lack of co-localization between AgDscam and Saccharomyces cerevisiae is indicative of interaction specificity ( Figure 4A; d1–d3).

AgDscam-depleted immune competent Sua5B cells and control cells treated with *GFP dsRNA* were co-incubated with heat-inactivated fluorescently labeled E. coli or S. aureus for 30 min to determine their phagocytic index as the number of cells containing fluorescent bacteria against the total ( Figure 4C). Western blot analysis showed that *AgDscam dsRNA* treatment of Sua5B cells effectively abolished AgDscam protein to 82% in the cell line ( Figure 4B). RNAi-mediated depletion of AgDscam decreased the phagocytic capacity of the Sua5B cells by about 60% for E. coli and 55% for *S. aureus,* compared to *GFP dsRNA-*treated control cells ( Figure 4C). These effects on phagocytosis were comparable to those shown for the A. gambiae Tep1 protein in a similar cell line and slightly higher than those observed for D. melanogaster Dscam in haemocytes [ 18, 49]. S. cerevisiae yeast cells were not effectively phagocytosed by the Sua5B cell line and only a few yeast cells could be found as loosely attached to the mosquito cells after co-incubation ( Figure 4A; d1–d3). Furthermore, yeast cells and AgDscam did not associate in in vitro binding assays (unpublished data). AgDscam is likely to be implicated in other defense mechanisms, in addition to phagocytosis, similarly to other immune factors [ 44, 49].

### AgDscam Can Associate with Bacteria

**Figure 5:**
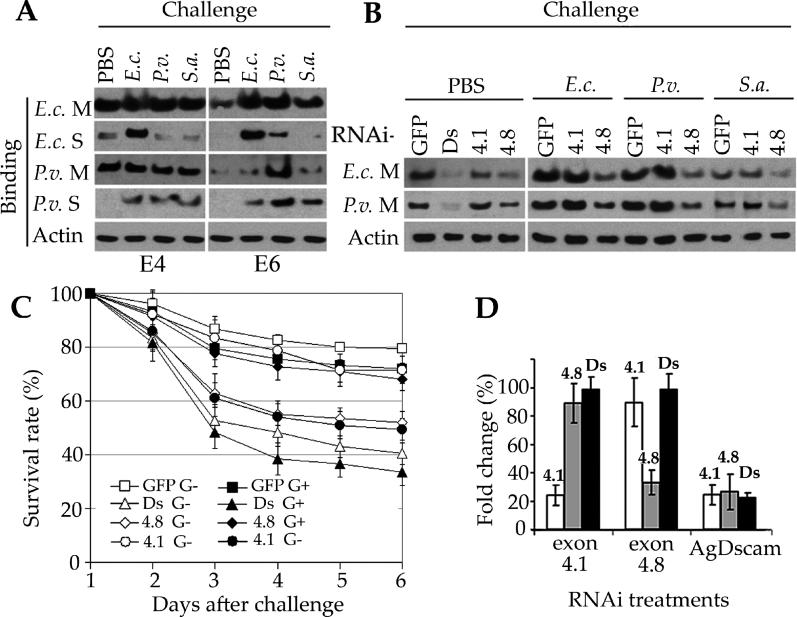
Pathogen-Induced AgDscam Splice Form Repertoires Have Increased Affinity to, and Defense Activity against, the Eliciting Pathogen (A) E. coli and P. veronii had increased affinity to both membrane-bound (M) and secreted (S) AgDscam splice form repertoires that were produced by Sua5B cells previously challenged with *E. coli (E.c.)* or *P. veronii (P.v.),* respectively, compared to cells challenged with *S. aureus (S.a),* or control cells treated with PBS. Similarly, P. veronii showed increased affinity to AgDscam splice forms that were produced by cells previously challenged with *P. veronii,* compared to cells challenged with E. coli. AgDscam was eluted from the bacteria surface with high salt concentrations (E4 = 400 mM, E6 = 500 mM +100 mM NH _4_Ac) after co-incubation with a cell line Sua5B membrane or secreted protein extract followed by washes with PBS. The lower degree of pathogen-specificity for the membrane-bound AgDscam form at the E4 elution was most likely due to excessive amount of bound protein to the bacteria surface. The last elution of the highest stringency was expected to release the AgDscam splice forms with the highest affinity and specificity to the bacteria. (B) Silencing of pathogen-induced specific isoform repertoires affect binding of AgDscam to the inducing pathogen. Silencing of the *E. coli-*induced exon 4.8-containing splice forms (4.8) resulted in decreased binding of AgDscam to E. coli and P. veronii compared to *GFP* and exon 4.1 *dsRNA*-treated cells, while silencing of the S. aureus-induced exon 4.1 (4.1) did not affect AgDscam binding to the two Gram-negative bacteria. This assay was done with both non-challenged cells (PBS) and cells that had been challenged 2 d after *dsRNA* treatments (GFP, Ds, 4.1, 4.8). Silencing of the total AgDscam (Ds) abolished AgDscam binding to both bacteria species. (C) Pathogen-induced splice form repertoires display increased defense activity to the eliciting pathogen. Upon selective silencing of the E. coli-induced splice form repertoire containing exon 4.8, mosquito's survival rate after G ^−^ *(E. coli)* challenge was significantly lower than that for G ^+^ *(S. aureus)* challenge (2-way ANOVA, *p <* 0.05). Conversely, selective silencing of the S. aureus-induced splice form repertoire containing exon 4.1 rendered the mosquitoes more sensitive to challenge with S. aureus (2-way ANOVA, *p <* 0.05). *GFP dsRNA*-treated mosquitoes challenged with E. coli (GFP G−) or S. aureus (GFP G+), total *AgDscam dsRNA*-treated mosquitoes challenged with E. coli (Ds G ^−^) or S. aureus (Ds G ^+^), exon 4.1 *dsRNA*-treated mosquitoes challenged with E. coli (4.1 G ^−^) or S. aureus (4.1 G ^+^), exon 4.8 *dsRNA*-treated mosquitoes challenged with E. coli (4.8 G ^−^) or S. aureus (4.8 G ^+^). (D) RTqPCR validation of RNAi gene-silencing efficiency and specificity. *dsRNA*s or *siRNA*s were used to target and specifically silence all AgDscam transcripts (AgDscam) and exon 4.1-, and exon 4.8-containing transcripts (displayed on the *x*-axis). The efficacy of silencing was assayed by RTqPCR with the primers specific for exon 4.1 (4.1), exon 4.8 (4.8), or the constant Dscam (Ds) for determination of the respective transcript abundance in the different gene-silenced samples and in a control *GFP dsRNA*-treated mosquito sample. The fold change of the expression is presented as the percentage (%) change compared to the *dsGFP*-treated control samples. cDNA template amounts from the different samples were normalized through amplification of an A. gambiae ribosomal S7 gene fragment as previously described [ 30]. Standard error bars from the three replica assays are presented.

The influence of AgDscam on the mosquito's immune defense could either reflect an indirect effect due to impairment of normal immune cell function, or indicate a direct implication in the defense against pathogens. The implication of AgDscam in phagocytosis and the adhesive nature of AgDscam suggest a receptor-like function in mediating defense upon interaction with microbes. Antibodies that specifically bind to the extracellular Ig domains of the D. melanogaster Dscam interfered with phagocytosis, and recombinant Dscam was shown to engage in splice form–specific direct interaction with microbes [ 18]. An in vitro bacterial binding assay based on the incubation of bacteria with immune cell membrane or soluble protein extracts was utilized to test the affinity between bacteria and AgDscam [ 51]. AgDscam could be eluted from the surface of E. coli at high salt concentrations after incubation of the bacteria with the immune cell protein extracts and several subsequent washes ( Figure 5A). The mediation of this interaction by other opsonins and potential adapter proteins is unlikely. Proteins extracted from solubilized membranes were used for the binding assays after exclusion of the secreted soluble protein fractions that would have contained such factors. The AgDscam gene produces both membrane-bound and soluble isoforms, and the fruit fly Dscam has been shown to be engaged in homophilic interactions, allowing Dscam molecules with identical Ig exon representations to interact [ 18, 52]. This could potentially serve as a mechanism of opsonizing pathogens with soluble Dscam splice forms and present them to cells expressing membrane-bound forms with the same exon representations.

### Immune Challenge Produces Anti-Pathogen-Specific AgDscam Splice Form Repertoires

Alternative splicing allows *Dscam* to produce a broad repertoire of receptors and thereby increase the probability of recognizing and defending against a broad spectrum of pathogens [ 33]. The infection-responsive splicing patterns of *AgDscam* suggested that certain splice forms were likely to be more specifically adapted for defense against the pathogens that elicited their expression ( Figure 1C). Indeed, the affinity of immune challenge-responsive *AgDscam* splice forms to microbes correlated with their induction specificity; infection-responsive regulation of *AgDscam* splicing resulted in the production of receptor molecules that had increased affinity to the infectious organism ( Figure 5A). For instance, both membrane-bound and secreted AgDscam splice form repertoires produced by cells challenged with the Gram-negative bacteria E. coli and P. veronii have a higher affinity to E. coli or P. veronii than AgDscam splice form repertoires that were produced by cells challenged with Gram-positive bacteria *S. aureus,* or non-challenged cell lines. This is likely relating to more similar exon representation patterns induced by the two Gram-negative bacteria compared to that induced by the Gram-positive bacterium S. aureus ( Figures 1C and 5A). The specificity of splice form repertoires' affinity to the different bacteria is obviously not comparable to that of antibodies, suggesting that even non-infection-responsive splice forms can associate with pathogens but at a lower affinity. This was also indicated by the degree of antimicrobial activity specificity for different splice form repertoires (discussed below). To further validate the specificity of splice form–specific pathogen associations, similar binding assays were performed in conjunction with selective silencing of specific splice form repertoires through RNAi targeting of S. aureus-induced exon 4.1 and *E. coli-* and P. veronii-induced exon 4.8 ( Figure 5B). Selective depletion of exon 4.8-containing splice forms resulted in decreased binding of AgDscam to E. coli and *P. veronii,* while selective depletion of exon 4.1-containing splice forms did not affect binding to these two Gram-negative bacteria. The in vitro properties of S. aureus did not allow interaction assays with AgDscam, which frequently generated weak associations with the protein in the in vitro assays. Splice form–specific and direct interaction has also been shown for recombinant D. melanogaster Dscam molecules to bacteria [ 18].

The significance of this splice form pathogen–association specificity for immune defense was demonstrated by the differential resistance patterns of mosquitoes to the different pathogens upon selective silencing of specific *AgDscam* splice form repertoire mRNAs [ 53]. Similarly to the cell line, exon 4.1 was specifically induced by the Gram-positive bacterium S. aureus and exon 4.8 by the Gram-negative bacterium E. coli in adult mosquitoes ( Figure S2). When the S. aureus challenge- induced exon 4.1-containing AgDscam splice forms were selectively silenced through targeting with a specific exon 4.1 *dsRNA,* the mosquitoes became significantly more sensitive to S. aureus infection compared to infection with E. coli (2-way analysis of variance (ANOVA), *p <* 0.05) ( Figure 5C). Similarly, when the E. coli infection-induced exon 4.8 was targeted with a specific exon 4.8 *dsRNA,* the mosquitoes became significantly less resistant to E. coli infection compared to S. aureus infection (2-way ANOVA, *p <* 0.05) ( Figure 5C). The effect of exon 4.1 silencing on E. coli resistance and exon 4.8 silencing on S. aureus resistance is likely to be attributed to a certain degree of cross-hybridization between the specific *dsRNA*s and non-target exons due to their high sequence identity and low affinity of the non-specific exons to each bacterium ( Figures 1C, 5C, 5D, and S1) [ 27, 53]. Even some of the constitutively expressed splice forms still possess a certain degree of affinity to, and activity against, the pathogen ( Figure 5B). Furthermore, the RNAi-targeted exons only represent a subset of the elicitor-induced splice forms that mediate defense, and the effect of single exon silencing is therefore only partial. The E. coli and S. aureus strains used in this study are most likely not natural pathogens to which A. gambiae mosquitoes are exposed to in the field, and A. gambiae may therefore not have evolved a highly specific AgDscam splice form for these bacteria. Through this mechanism, the mosquito can more efficiently target specific pathogens by selective production of a limited range of PRRs with increased affinity and defense activity, instead of producing a very broad repertoire of random splice forms that would only contain a small proportion of pathogen-specific receptors.

### Conclusions

This study establishes AgDscam as an essential hypervariable receptor of the insect immune surveillance system, with the capacity of phenotypic plasticity to different spectra of microbial exposure [ 27, 54]. The exceptional degree of AgDscam variability provides the potential to increase the mosquito's pattern recognition repertoire by 15,960 potential different adhesive Ig domain combinations (considering the two alternative transmembrane domain exons that may not influence binding specificity). The broad range of constitutively expressed AgDscam receptor molecules will narrow down to a more specific receptor repertoire that is more compatible with the infectious organism upon immune activation. *AgDscam* produces pathogen challenge-specific splice form repertoires enriched with receptor molecules of increased affinity and defense specificity to the eliciting pathogen. Induction of pathogen, or pathogen class-specific antimicrobial peptides and other immune factors, is controlled by immune signaling pathways, such as Toll and Imd in D. melanogaster [ 4, 15]. Splicing factors, that will determine AgDscam Ig exon prevalence, are also likely to be regulated by the same or similar pathways, allowing the insect to produce different pathogen, or pathogen class-specific receptors from the same gene [ 41]. Preliminary assays have shown altered AgDscam splicing upon RNAi-mediated depletion of a Rel family transcription factor Rel2 prior to LPS challenge of the Sua5B cell line [ 32] (Dimopoulos lab, unpublished data). The insect can in this way achieve a tremendous increase of its PRR repertoire and thereby more efficiently cope with a broad range of pathogens. Dscam represents a component that is shared between the nervous system and the innate immune system, both requiring a high degree of pattern recognition specificity. As suggested by Boulanger and Shatz [ 55, 56], “the brain and the immune system speak a common biological language.” The mammalian *Dscam* gene does not undergo the same degree of alternative splicing as the insect homologs and can only produce three different mRNA forms [ 24]. This may suggest a more specialized function of Dscam in the nervous system of vertebrates that use their antibodies for pathogen recognition. *Dscam* Ig exon duplications in the insect genomes and their alternative splicing may mainly have evolved to provide diversity for broad spectrum pathogen recognition as well as for neuronal wiring [ 20, 27]. A. gambiae is an important vector of disease, and a detailed understanding of its innate immune system and how it recognizes *Plasmodium* could be utilized for the development of alternative malaria control strategies.

## Materials and Methods

### Mosquito rearing.

*A. gambiae* Keele strain mosquitoes were maintained on sugar solution at 27 °C and 70% humidity with a 12-h light/dark cycle according to standard procedures [ 57].

### Immune challenge in adult mosquito and cell line.

Approximately 10 ^7^ heat-inactivated E. coli DH5α, *P. veronii, S. aureus,* and Beauveria bassiana spores were separately added to one well of 6-well plate (Corning) with about 10 ^6^ of A. gambiae Sua5B cells [ 30]. A lower dosage, 10 ^6^ CFU/10 ^6^ cells was also tested. Sterile PBS was added to controls. LPS from Pseudomonas aeruginosa serotype 10 (Sigma-Aldrich, St. Louis, Missouri, United States) and PG from S. aureus (Sigma-Aldrich) were dissolved in water and added to cells at 10 μg/ml [ 32, 58]. After 12 h or 18 h of challenge, the cells were subjected three times to washing with PBS, and total RNA was extracted with RNeasy kit (Qiagen, Valencia, California, United States). *P. berghei GFP* parasites and P. falciparum infections were performed as previously described [ 46, 59]. Total RNA was extracted 24 h after feeding on infected and non-infected mouse or blood. Infection levels were determined by counting the oocysts numbers in 20 or more mosquitoes. For P. berghei infection, mosquitoes were maintained at 21 °C for 13 d before dissection; for P. falciparum infection, mosquitoes were maintained at 24 °C for 8 d.

### Real-time quantitative PCR.

The total RNA samples were treated with Turbo DNase (Ambion, Austin, Texas, United States) and reverse-transcribed using Superscript III (Invitrogen, Carlsbad, California, United States) with random hexamers. Real-time quantitative PCR (RTqPCR) was performed using the QuantiTect SYBR Green PCR Kit (Qiagen) and ABI Detection System ABI Prism 7000 (Columbia, Maryland, United States). All PCR reactions were performed in triplicate; to check for specificity of the PCR reactions, melting curves were analyzed for each data point. The relative levels of expression of *AgDscam* exon 4 members were determined by normalizing cDNAs using ribosomal S7 gene and constitutive non-spliced Dscam exons. Raw data were presented in Table S1. The significance of spliced exon regulation was determined based on a *t*-test, *p* < 0.05, through comparison to the regulation of a constant non-spliced Dscam exon ( Table S1). Primer sequences, in 5′ to 3′ orientation, used in the real-time PCR analysis were: S7: A TCCTGGAGCTGGAGATGAAC, B: GACGGGTCTGTACCTTCTGG; *AgDscam*: Exon 3: A: AGTTCGGGTCGGTGATTTC, Exon 4.1: B: CATGCGTTTCCTCCTCATCG, Exon 4.2: B: GGTGGAGTTGGCATAGAAGC, Exon 4.3: B: CGGTTGTCGAGCTGTTGATA, Exon 4.4: B: CGAAGCTCGATGCATTGTAA, Exon 4.5: B: TTTCCGAGTGAACAAGCTCA, Exon 4.6: B: CGACTGGACAAGCTCGTTG, Exon 4.7: B: GTGGTACTCGTTCCCGTCAC, Exon 4.8: B: AATCCTGCTCTCCGCTATGA, Exon 4.9: B: TGGTCGCTTGGTTGTACTCC, Exon 4.10: B: CTCTGCGCCCGGATAGTATT, Exon 4.11: B: TCATCCGCATCGATCATTAC, Exon 4.12: B: CATCCGTGACGGGAAGATAC, Exon 4.13: B: AACCAGCCGGTAACGCTAAT, Exon 4.14: B: GCGGTCCACTCAATGATCTC, Exon 17: A: GAACGATGGGATCGAGCA, Exon 18/19: B: TGCGTGTGTAACGTCGGTAT.

### Gene silencing in adult A. gambiae and cell line.

Sense and antisense RNAs were synthesized from PCR-amplified gene fragments using the T7 MEGAscript kit (Ambion). About 69 nl *dsRNA*s (3 μg/μl) in water was introduced in the thorax of cold-anesthetized 4-d-old female mosquitoes by a nano-injector (Nanoject, Drummond, Broomell, Pennsylvania, United States) with glass capillary needles according to established methodology [ 43]. *GFP dsRNA* was used as control. Confirmation of *AgDscam* silencing was done with RT-qPCR. The exon-specific RNAi assays were done as previously described and the entire sequence of exons 4.1 and 4.8 were represented by the *dsRNA*s [ 43, 53]. Assays yielding similar results were also performed with siRNA. For gene silencing in the Sua5B cell line, cells were re-suspended as 1 × 10 ^6^ cells in 0.5 ml of Schneider's media and incubated with 20 μg of *dsRNA* for 30 min with gentle agitation. Cells were *dsRNA*-treated for 6 d and then plated on glass cover slips 24 h prior to phagocytotic assays [ 49, 60].

### Bacterial and malaria infection of adult mosquitoes.

Gram-positive bacteria S. aureus and Gram-negative bacteria E. coli DH5α were cultured in LB broth overnight, washed three times with (PBS), and re-suspended in PBS. At 4 d after *dsRNA* treatments the anaesthetized mosquitoes were injected with 69 nl of either S. aureus (OD _600_ = 0.4) or E. coli (OD _600_ = 0.1) into the haemolymph. Injections were done using a microcapillary Nanoject II injector (Drummond). Control *dsRNA-*treated mosquitoes were injected with 69 nl sterile PBS. Dead mosquitoes were counted and removed daily over a 6-d period. The results shown here were representative of 50 mosquitoes for each treatment and at least three independent experiments per tested group. A Kaplan Meier survival analysis was used to determine the median survival time, and a 2-way ANOVA for the significance of the treatments. For P. bergheii infection, the mosquitoes were fed on the same infected mouse 4 d after *dsGFP* and *AgDscam dsRNA* treatment [ 46, 47]. Unfed mosquitoes were removed 24 h post-infection, and the rest were left in a 21 °C incubator for 13 d. Mosquito midguts were dissected and the numbers of oocysts were determined using a fluorescent microscope (Olympus, Tokyo, Japan). Each assay was done with at least 25 mosquitoes, and the results shown here were the pool of four independent experiments with equal number of midguts from each experiment pooled [ 46].

### Bacteria isolation from gene-silenced mosquitoes and CFU enumeration and determination.

3 d after the *dsRNA* treatment, live mosquitoes were cold-anesthetized and surface-sterilized with 70% ethanol and subsequently washed three times with PBS. The efficacy of the sterilization was determined by exposing the sterilized mosquito to a LB plate for 15 min and subsequently incubated in the plate for 2 d in 27 °C to monitor the growth of bacteria. The haemolymph from sterilized mosquitoes were collected with a capillary needle and diluted in sterile PBS. The CFU was determined by plating the dilutions on LB plates followed by incubation at 30 °C for 2 d. The morphology of different bacteria was determined and each bacterium was purified by re-streaking a single colony on the LB plate three times. Each experiment was performed with the haemolymph of two mosquitoes and results shown were representative of 12 independent experiments. 16s rDNA from different bacteria species were amplified by PCR as described using primers 27f and 1492r [ 61]. 16s rDNA PCR products were sequenced, and the bacterial species were determined using BLAST Sequence Similarity Search [ 62].

### Phagocytic assays.

Gene-silenced cells were washed with PBS twice and about 10 ^7^ fluorescein conjugates of E. coli or S. aureus (Molecular Probes, Eugene, Oregon, United States) were added to 10 ^6^ Sua5B cells and incubated at room temperature for 30 min with gentle rocking. Internalized microbes were detected as described previously at 30 min after co-incubation with fluorescein-conjugated bacteria. Ethidium bromide at 150 μg/ml was used as a quencher [ 49, 60]. The phagocytic index was determined as the ratio of the number of cells containing fluorescent bacteria against the total number of cells in each field. For each assay, at least 20 fields were included, with as least three independent replicas. Student *t-*test was used to address the statistical significance of treatments.

### Immunofluorescence microscopy.

Sua 5B cells were seeded on glass cover slips placed in 6-well tissue culture plates (Corning) yielding half-confluent cell layers. After incubation at 27 °C for 3 d, about 10 ^7^ FITC conjugates of E. coli (Molecular Probes) or S. cerevisiae were added to 10 ^6^ mosquito cells on cover slips and incubated at room temperature for 30 min with gentle rocking. Immuno-fluorescence microscopy assays were done according to previously described procedures with some modifications [ 30]. The cover slips were washed three times with PBS and then fixed in PBS/4% formaldehyde for 20 min. Cover slips were subjected to one PBS wash and then exposed for 2 min to 0.2% Triton X-100 in PBS followed by two additional PBS washes. After blocking with 1% bovine serum albumin in PBS for 2 h, cover slips were incubated with the *Drosophila* anti-Dscam serum (D-cy) or pre-immune serum diluted 1:500 in 1% bovine serum albumin/PBS overnight at 4 °C. Samples were washed three times in PBS and incubated for 1 h with a fluorescein Rhodamine-conjugated anti-rat IgG antibody (Molecular Probes) diluted 1:500 in 1% bovine serum albumin/PBS. After the final PBS washes, the cover slips were mounted with Prolong Antifade kit with DAPI (Molecular Probes). Ten sequential optical sections of 1 μm each were collected and only one optical section was shown. Cover slips were sealed with nail polish and subjected to a Zeiss 510 system-based confocal microscopy. An A. gambiae Dscam antibody raised against a short peptide (RIRQLPEGSLFIKDC) of the non-spliced *AgDscam* Ig domain 12 exon was also used and generated comparable results (not shown).

### In vitro bacteria AgDscam binding assays.

About 10 ^6^ Sua 5B cells were first challenged with 10 ^7^ heat-inactivated and PBS-washed *E. coli, S. aureus,* and P. veroii for 24 h. Membrane and soluble proteins were separately prepared with the ProteoExtract Membrane Protein Extraction Kit (Calbiochem, San Diego, California, United States) according to the manufacturer's instructions after removal of non-phagocytosed bacteria through washes. The amounts of protein for the binding assays were determined and normalized with a BCA protein assay kit (Pierce, Rockford, Illinois, United States) and the actin protein content using a specific actin antibody (Sigma). Bacterial binding assays were done as described previously with some modifications [ 51]. Briefly, 10 ml of late logarithmic-phase culture of *E. coli, S. aureus,* and P. veronii were centrifuged at 8,000 rpm 2 min, and re-suspended in 1/10 of the original volume in 0.2 M NaCl. Bacteria were then inactivated by 10% acetic acid and incubated at room temperature for 10 min. The solution was neutralized with five volumes of 1 M Tris-HCl [pH 8.0] and bacteria were washed three times with PBS followed by re-suspending in 1/20 of the original volume in 10 mM Tris-HCl [pH 8.0]. For the binding assay, equal amounts of these acid-inactivated bacterial suspensions (2.0 × 10 ^10^ CFU) were added to 1 ml of same concentration of either membrane or soluble protein extract from each bacterial challenge or control. The mixture was then incubated at 4 °C for 1 h to overnight under gentle agitation. Samples were then centrifuged for 5 min at 8,000 *g* at 4 °C and the supernatant was removed. The bacteria pellet was pre-washed in 200 μl of 10 mM Tris-HCl [pH 8.0]. Bacteria-binding proteins were eluted with 200 μl of 10 mM Tris-HCl [pH 8.0] containing a gradient of increasing amounts of NaCl (0.1, 0.2, 0.3, and 0.4 M NaCl). A final elution was performed using 200 μl of 0.5 M NaC1/0.1 M NH _4_Ac [pH 5.0]. Finally, 15 μl of eluted proteins from different microbe binding assays was subjected to 4%–12% SDS-PAGE (Invitrogen Novex Tris-Gycine Gel) and AgDscam was detected using Western blot analysis. The *Drosophila* Dscam antibodies raised against the cytoplasmic domain (D-cy) and extracellular domain (Ig 1–4) (D-ex1) were used at 1:1000 dilutions for membrane and soluble protein assays, respectively [ 18]. The eluted AgDscam was most frequently processed to shorter peptides confirmed as AgDscam by RNAi-mediated depletion [ 18]. An A. gambiae Dscam antibody raised against a short peptide (RIRQLPEGSLFIKDC) of the non-spliced *AgDscam* Ig domain 12 exon was also used and generated comparable results (not shown).

## Supporting Information

Figure S1 Phylogenetic Tree of Nucleotide Sequences Coding for *AgDscam* Ig Domain Exons 4, 6, and 10 Splicing Forms
Full-length DNA sequences of the *AgDscam* Ig domain exons of exon cassettes 4, 6, and 10 were aligned using the Clustal X program, and cladograms were constructed by neighbor-joining analysis and displayed through Treeview.
(18.1 MB TIF)

Figure S2 *AgDscam* Gene Organization
(A) The variable Ig domain exon cassettes 4, 6, and 10 are displayed as Ig4(14) (red), Ig6(30) (blue), and Ig10(38) (green), each containing 14, 30, and 38 exons, respectively. The transmembrane domain exons (tm) 11–13 are displayed in pink color, and non-spliced exons are displayed in black.
(B) Percentage identity of AgDscam exons to the D. melanogaster Dscam exons at the amino acid level is displayed. Phylogenetic relations between AgDscam Ig exons are presented in Figure S1.
(C) Differences in *AgDscam* exon 4 transcript representations between bacterial- *(E. coli, P. veronii, S. aureus),* fungus- *(B. bassiana),* LPS-, and PG-challenged cell line Sua5B and a non-challenged cell line at 12 h after challenge, as well as the expressions of cells challenged with E. coli at a 10-fold lower dosage (LD) and challenged with E. coli and S. aureus at a later time point of 18 h, are presented. Adult 4-d-old mosquitoes challenged with *E. coli, S. aureus,* and B. bassiana at 12 h are coded with *Ag* in front of the corresponding elicitors. *Plasmodium- (P. berghei* and *P. falciparum)* infected midguts *(Ag)* and non-infected blood-fed midguts at 24 h after ingestion. The numbers (“4.1” –“4.14”) at the bottom of the figure indicate the exon numbers of exon cassette 4. The expression patterns upon different elicitors challenge were clustered with Cluster and TreeView software based on the value of correlation coefficient, where the cluster tree is presented on the left part. Expression was determined with RTqPCR analyses in three replica assays. The fold difference in exon representation (ratio) between challenged and naive samples from three replicates were determined in normalized cDNAs, and expression ratios are displayed in a color scheme where red indicates a higher representation and green indicates a lower representation of exons in challenged samples compared to naïve samples. Black was indicative of a lack of infection-responsive regulation. Normalization was done with expression analysis of non-spliced *AgDscam* exons and a ribosomal S7 gene. The cut-off value for gene expression at 95% confidence level, the RTqPCR efficiencies for each pair of exon primers, expression data values, and standard errors are presented in Table S1.
(20.4 MB TIF)

Figure S3 Kaplan-Meier Survival Analysis of *AgDscam dsRNA*-Treated Mosquito Survival Probability after Challenge with S. aureus and E. coli Compared to Control *GFP dsRNA*-Treated (GFP) Mosquitoes
Total sample size for each treatment is 150. Kaplan-Meier analysis showed the survival rate for *AgDscam-*treated mosquitoes challenged with either S. aureus or E. coli is significantly lower than that from the *GFP dsRNA*-treated control mosquitoes ( *p* < 0.0001). The median survival time for *AgDscam dsRNA-*treated mosquitoes was 2.5 d after challenge with S. aureus and 3 d with E. coli. Mosquitoes that remained alive at the end of the experiment were excluded when comparing the median survival time.
(8.4 MB TIF)

Figure S4 Effect of RNAi Gene Silencing of *AgDscam* on Parasite Development in Mosquito Midgut
*CTRL:* GFP *dsRNA* control; *AgDscam KD: AgDscam-*silenced mosquito. Control and *AgDscam-*silenced mosquitoes were blood fed from the same mouse infected with the P. berghei GFP, oocysts morphology was observed, and oocysts numbers were counted with fluorescent microscope at day 13 after feeding.
(5.3 MB TIF)

Table S1 RTqPCR Analyses of AgDscam Exon 4 Transcripts upon Challenges (162 KB DOC)

### Accession Numbers

The GenBank ( http://www.ncbi.nlm.nih.gov/Genbank) accession number for *Bacterium* HPC1068 is DQ413250.

## Abbreviations

ANOVA: analysis of variance
CFU: colony-forming unit
Ig: immunoglobulin
LPS: lipopolysaccharide
PG: peptidoglycan
PRR: pattern recognition receptor
RNAi: RNA interference
RTqPCR: real-time quantitative PCR
