# Supplementary material for: AgDscam, a Hypervariable Immunoglobulin Domain-Containing Receptor of the Anopheles gambiae Innate Immune System
Source: PLoS Biol. 2006 Jun 20;4(7):e229. doi: 10.1371/journal.pbio.0040229 (PMC1479700; doi:10.1371/journal.pbio.0040229)
Supplement: Supplementary file 1 [file pbio.0040229.st001.doc]

**Supporting Information Table S1.**

Real-time quantitative PCR (QRT-PCR) analyses of *AgDscam* exon 4 transcripts upon challenges.

| **exon Ig** |  | **4.1** | **4.2** | **4.3** | **4.4** | **4.5** | **4.6** | **4.7** | **4.8** | **4.9** | **4.10** | **4.11** | **4.12** | **4.13** | **4.14** | |
| --- | --- | --- | --- | --- | --- | --- | --- | --- | --- | --- | --- | --- | --- | --- | --- | --- |
| QRT-PCR efficiency |  | 0.9 | 0.91 | 0.9 | 0..92 | 0.91 | 0.9 | 0.91 | 0.9 | 0.91 | 0.9 | 0.9 | 0.91 | 0.9 | | 0.91 |
| ***E. coli/* PBS (LD)** | fold ^a^ | 0.47 | 1.38 | 0.80 | 1.15 | 0.59 | 0.79 | 1.44 | 1.65 | 1.11 | 0.56 | 0.92 | 1.03 | 1.24 | | 1.53 |
|  | log2 ^b^ | -1.08 | 0.47 | -0.32 | 0.21 | -0.76 | -0.35 | 0.53 | 0.73 | 0.15 | -0.85 | -0.11 | 0.05 | 0.31 | | 0.61 |
|  | S.E. ^c^ | 0.11 | 0.12 | 0.03 | 0.37 | 0.26 | 0.18 | 0.11 | 0.07 | 0.05 | 0.09 | 0.33 | 0.24 | 0.15 | | 0.16 |
| ***E. coli/* PBS** | fold | 0.25 | 1.45 | 2.71 | 1.80 | 0.51 | 1.18 | 1.37 | 2.53 | 0.52 | 0.70 | 0.89 | 0.55 | 2.01 | | 5.21 |
|  | log2 | -1.99 | 0.54 | 1.44 | 0.85 | -0.98 | 0.24 | 0.45 | 1.34 | -0.95 | -0.51 | -0.17 | -0.85 | 1.01 | | 2.38 |
|  | S.E. | 0.23 | 0.10 | 0.09 | 0.21 | 0.13 | 0.04 | 0.08 | 0.22 | 0.12 | 0.11 | 0.04 | 0.22 | 0.21 | | 0.10 |
| ***E. coli/* PBS (18h)** | fold | 0.37 | 3.73 | 1.72 | 1.56 | 0.80 | 1.45 | 1.47 | 2.27 | 1.12 | 1.19 | 0.95 | 0.76 | 2.57 | | 3.15 |
|  | log2 | -1.44 | 1.90 | 0.79 | 0.64 | -0.32 | 0.54 | 0.56 | 1.19 | 0.16 | 0.25 | -0.07 | -0.40 | 1.36 | | 1.66 |
|  | S.E. | 0.11 | 0.35 | 0.28 | 0.18 | 0.19 | 0.20 | 0.21 | 0.31 | 0.05 | 0.09 | 0.11 | 0.04 | 0.25 | | 0.31 |
| ***P. veronii/* PBS** | fold | 0.40 | 4.20 | 2.48 | 2.64 | 0.92 | 0.91 | 1.83 | 3.86 | 0.63 | 0.34 | 0.76 | 1.01 | 3.12 | | 2.25 |
|  | Log2 | -1.33 | 2.07 | 1.31 | 1.40 | -0.12 | -0.13 | 0.87 | 1.95 | -0.66 | -1.57 | -0.40 | 0.01 | 1.64 | | 1.17 |
|  | S.E. | 0.30 | 0.40 | 0.20 | 0.33 | 0.03 | 0.04 | 0.28 | 0.33 | 0.18 | 0.31 | 0.09 | 0.02 | 0.32 | | 0.25 |
| ***S. aureus/* PBS** | fold | 2.39 | 0.95 | 0.72 | 0.64 | 1.74 | 0.88 | 0.28 | 0.35 | 0.87 | 1.79 | 1.09 | 2.00 | 2.75 | | 2.33 |
|  | log2 | 1.26 | -0.07 | -0.47 | -0.65 | 0.80 | -0.19 | -1.82 | -1.51 | -0.20 | 0.84 | 0.13 | 1.00 | 1.46 | | 1.22 |
|  | S.E. | 0.28 | 0.04 | 0.10 | 0.14 | 0.18 | 0.08 | 0.40 | 0.33 | 0.04 | 0.18 | 0.03 | 0.22 | 0.32 | | 0.27 |
| ***S. aureus/* PBS (18h)** | fold | 2.17 | 1.20 | 1.40 | 0.75 | 1.44 | 0.66 | 0.43 | 0.36 | 1.51 | 2.45 | 1.85 | 2.18 | 1.74 | | 2.08 |
|  | log2 | 1.12 | 0.26 | 0.49 | -0.42 | 0.52 | -0.59 | -1.23 | -1.46 | 0.60 | 1.30 | 0.89 | 1.13 | 0.80 | | 1.06 |
|  | S.E. | 0.21 | 0.13 | 0.09 | 0.14 | 0.25 | 0.08 | 0.09 | 0.24 | 0.18 | 0.29 | 0.19 | 0.25 | 0.22 | | 0.31 |
| ***B. bassiana/* PBS** | fold | 2.05 | 0.75 | 1.13 | 1.12 | 1.81 | 1.53 | 1.07 | 2.13 | 1.04 | 0.65 | 2.54 | 1.51 | 1.18 | | 1.03 |
|  | log2 | 1.04 | -0.41 | 0.18 | 0.16 | 0.86 | 0.61 | 0.10 | 1.09 | 0.06 | -0.63 | 1.35 | 0.59 | 0.24 | | 0.05 |
|  | S.E. | 0.25 | 0.06 | 0.23 | 0.43 | 0.23 | 0.16 | 0.27 | 0.18 | 0.03 | 0.11 | 0.29 | 0.02 | 0.06 | | 0.14 |
| **LPS*/* PBS** | fold | 0.58 | 2.22 | 4.41 | 3.78 | 1.31 | 1.37 | 0.93 | 3.56 | 1.99 | 1.93 | 1.26 | 3.27 | 3.12 | | 1.87 |
|  | log2 | -0.78 | 1.15 | 2.14 | 1.92 | 0.39 | 0.45 | -0.11 | 1.83 | 0.99 | 0.95 | 0.33 | 1.71 | 1.64 | | 0.90 |
|  | S.E. | 0.17 | 0.15 | 0.35 | 0.20 | 0.31 | 0.24 | 0.00 | 0.37 | 0.11 | 0.13 | 0.28 | 0.15 | 0.41 | | 0.08 |
| **PG*/* PBS** | fold | 1.31 | 1.57 | 2.06 | 1.48 | 1.57 | 1.58 | 0.84 | 1.06 | 1.85 | 0.74 | 1.41 | 2.06 | 1.70 | | 0.71 |
|  | log2 | 0.39 | 0.65 | 1.04 | 0.56 | 0.65 | 0.66 | -0.24 | 0.09 | 0.89 | -0.44 | 0.50 | 1.04 | 0.77 | | -0.50 |
|  | S.E. | 0.09 | 0.33 | 0.19 | 0.15 | 0.18 | 0.03 | 0.05 | 0.05 | 0.06 | 0.09 | 0.07 | 0.10 | 0.35 | | 0.09 |
| ***Ag E. coli/* PBS** | fold | 0.27 | 1.51 | 5.46 | 2.43 | 0.44 | 2.97 | 2.44 | 2.71 | 2.56 | 0.77 | 7.16 | 0.64 | 5.13 | | 3.86 |
|  | log2 | -1.89 | 0.59 | 2.45 | 1.28 | -1.18 | 1.57 | 1.29 | 1.44 | 1.36 | -0.38 | 2.84 | -0.65 | 2.36 | | 1.95 |
|  | S.E. | 0.14 | 0.22 | 0.18 | 0.02 | 0.01 | 0.11 | 0.11 | 0.02 | 0.23 | 0.17 | 0.33 | 0.17 | 0.02 | | 0.48 |
| ***Ag S. aureus/* PBS** | fold | 2.99 | 0.66 | 2.52 | 2.55 | 1.35 | 2.23 | 0.55 | 0.45 | 2.54 | 2.51 | 3.36 | 0.24 | 2.88 | | 1.25 |
|  | log2 | 1.58 | -0.60 | 1.34 | 1.35 | 0.43 | 1.16 | -0.85 | -1.14 | 1.34 | 1.33 | 1.75 | -2.08 | 1.53 | | 0.33 |
|  | S.E. | 0.08 | 0.29 | 0.12 | 0.12 | 0.66 | 0.06 | 0.14 | 0.10 | 0.30 | 0.20 | 0.37 | 0.35 | 0.17 | | 0.13 |
| ***Ag B. bassiana/*PBS** | fold | 1.32 | 0.88 | 1.26 | 1.19 | 0.62 | 1.03 | 0.70 | 2.05 | 0.93 | 2.96 | 0.52 | 0.70 | 1.03 | | 1.20 |
|  | log2 | 0.40 | -0.18 | 0.33 | 0.25 | -0.70 | 0.05 | -0.52 | 1.03 | -0.10 | 1.56 | -0.95 | -0.52 | 0.04 | | 0.26 |
|  | S.E. | 0.01 | 0.03 | 0.11 | 0.10 | 0.05 | 0.25 | 0.10 | 0.14 | 0.05 | 0.25 | 0.24 | 0.07 | 0.21 | | 0.21 |
| ***P.berghei/* BF** | fold | 0.29 | 2.19 | 1.97 | 2.23 | 1.84 | 1.30 | 1.27 | 1.86 | 2.86 | 1.53 | 2.82 | 1.66 | 1.52 | | 3.00 |
|  | log2 | -1.77 | 1.13 | 0.98 | 1.16 | 0.88 | 0.38 | 0.34 | 0.90 | 1.52 | 0.61 | 1.50 | 0.73 | 0.60 | | 1.59 |
|  | S.E. | 0.76 | 0.24 | 0.11 | 0.23 | 0.27 | 0.05 | 0.19 | 0.19 | 0.22 | 0.06 | 0.37 | 0.34 | 0.29 | | 0.52 |
| ***P.falciparum/*BF** | fold | 4.24 | 0.36 | 0.86 | 1.60 | 3.58 | 0.33 | 1.22 | 0.18 | 3.24 | 2.78 | 3.77 | 1.59 | 0.71 | | 0.23 |
|  | log2 | 2.08 | -1.48 | -0.21 | 0.68 | 1.84 | -1.60 | 0.29 | -2.46 | 1.70 | 1.48 | 1.91 | 0.67 | -0.49 | | -2.13 |
|  | S.E. | 1.05 | 0.02 | 0.08 | 0.57 | 0.46 | 0.85 | 0.15 | 1.12 | 0.33 | 0.47 | 0.19 | 0.36 | 0.86 | | 1.36 |

^a^ fold: fold changes (ratio) of challenged vs un-challenged; ^b^ log2: log2 transformed of ratio; ^c^ S.E.: standard error of three replicas; ^d^ BF: mouse blood fed; ^e^ BF: human blood fed. Numbers in red indicates the significant up-regulation and green indicates the significant down regulation at 95% confidence interval based on *t-test* p value.
